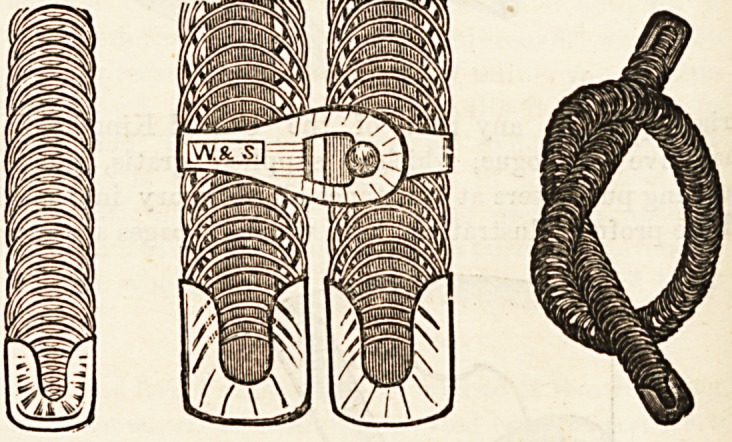# "The Hospital" Nursing Mirror

**Published:** 1899-07-15

**Authors:** 


### "The Hospital" Nursing Mirror.

Being the Nursing Section of "The Hospital."

[Contributions for this Section of "The Hospital" should be addressed to the Editor, The Hospital, 28 & 29, Southampton Street, Strand, London, W.C., and should have the word "Nursing" plainly written in left-hand top corner of the envelope.]

### Notes on News from the Nursing World.

### RECEPTION OF NURSES AT MARLBOROUGH HOUSE.

The most interesting function in the nursing world is to take place on Friday, July 21st, at half-past twelve p.m. We refer to the reception at Marlborough House by the Princess of Wales, as President of the Royal National Pension Fund, of nurses entitled to certificates. There will be a preliminary meeting in the grounds of Marlborough House at half-past ten on the same day, which every nurse who wishes to be at the presentation must attend. Nurses entitled to be present are policy-holders whose policies are numbered 4,001 to 7,250, both inclusive, unless the holders have been entitled to attend a previous reception under an earlier policy number. The reception is, therefore, likely to be a very large one. Her Royal Highness has been unable to give a long notice of her intention, and all the officials at the Pension Fund Office will in consequence be very busy next week. We feel sure that nurses will appreciate the position, and carefully study the instructions sent to them, with the view of saving unnecessary trouble. Nursing uniform is, of course, obligatory, and indoor uniform is recommended for all who generally wear it. District nurses, of whose costumes bonnets form an essential part, can wear them in place of caps if they like, but the cloaks should be omitted. As every nurse will wear the "Princess's Armlet," the sight will be exceedingly pretty. Matrons or superintendents of homes for nurses, or others who are able to accommodate nurses on the evening of the 20th, will greatly oblige by communicating with the Secretary of the Fund, 28, Finsbury Pavement, E.C., stating terms for bed and breakfast. The office of the Fund will be closed on the day of the ceremony.

### THE ROYAL BRITISH NURSING ASSOCIATION.

The vacancies on the general and executive councils of the Royal British Nurses' Association have been filled up as follows: On the General Council two medical men, Dr. Schofield and Dr. Owen Lankester; one matron, Miss Vernet (matron of the National Hospital for the Paralysed and Epileptic); and two nurses, Miss Alice Lemon (superintendent sister of the Biblewomen's and Nurses' Mission), and Miss Agnes Macvicar. On the Executive Committee four medical men, Dr. Richard Greene, Mr. Arthur Barker, Dr. Clement Godson, and Mr. Warrington Haward; three matrons, Miss Hull (matron of the Great Northern Central Hospital), Miss Anderson (matron of the St. Saviour's Home, Hendon), and Miss Vernet (matron of the National Hospital for the Paralysed and Epileptic); and three sisters and nurses, Miss Arnott (private nurse), Miss Honnor (St. John's House), and Miss Nesbit (St. George's Hospital).

### THE DEVELOPMENT OF NURSING IN GLASGOW.

The Glasgow and West of Scotland Co-operation for Trained Nurses has, since its formation in 1893, proved a great success, both in respect to the work done by the nurses and as regards the financial position of the institution. The co-operation now includes close upon one hundred fully trained nurses, whose professional qualifications are carefully investigated by a medical committee before they are admitted. In order to meet the constantly increasing requirements of the public, who more and more realise the value of good nursing, the executive committee now recognise that they must increase the staff to 150 nurses, otherwise the co-operation will fail to meet the demands made upon it. This they hope to do before next winter. The late Professor Joseph Coats, along with the present chairman of the executive committee, Dr. David Newman, took an active part in the formation and development of the co-operation. During last year the employment was so constant that the nurses earned from £70 to £85 a year.

### NURSING AT BUENOS AYRES.

The English nurses who are engaged at the Hospital Ingles, Buenos Ayres, have no reason to complain that things are not up-to-date. The hospital, which was erected in 1885, has 110 beds, and the wards are built apart on the pavilion system, connected by covered corridors. A new operating theatre has recently been added, containing modern furniture and appliances. The institution is entirely under the control of a non-resident medical officer, but he has a fully-trained qualified resident assistant. The indoor staff consists of a matron, with 18 nurses and three male attendants. There is also a dispenser, who is non-resident. Two of the nurses went out from England on contract; the others are trained in the hospital. Probationers enter for two years, without premium, receiving a small salary, increasing by degrees. At the end of that period they become assistant nurses, and are promoted to charge, if suitable, and a vacancy occurs. Sixteen of the beds are for private paying patients. The rule of the hospital is that patients in the general wards pay according to their circumstances, unless sent in by subscribers' ticket, or recommended as deserving cases.

### THE ALEXANDRA HOSPITAL.

The lady superintendent who has presided over the nursing destinies of the Alexandra Hospital for Children with Hip-joint Disease in the old, and later in the temporary, quarters of the hospital, is looking forward to occupying the adequate and convenient just-completed buildings which will be opened next week by the Prince and Princess of Wales. She intends beginning work with a sister, assisted by a nurse and three probationers, on each of the two floors, two out-patient sisters, two night nurses, and one in charge of the isolation flat, Unfortunately it has been found impossible to give every nurse a separate bedroom, and it has been thought better to put two nurses in one room than to cut up the available space into cubicles. The rooms are fairly large, well ventilated, warmed by radiators, and, judging from the samples of wardrobes and dressing-chests, will be prettily furnished. Probationers receive a two years' training at this hospital. Some of them become so much attached to the work that they remain for years. Not the least satisfactory result of the hospital's work is the number of perfect cures following the long, tedious illness and recovery. One boy is now playing football; some girls are earning their living as machinists. The cost of the new buildings is £20,000, and the secretary is anxiously looking out for handsome donations in order that the opening ceremony may find them free from debt.

### PRIZE DAY AT GUY'S.

Guy's Hospital presented an animated appearance on Friday—prize day and a delightful garden party being combined in a harmonious whole. The "Park" in the midst of the hospital buildings was gay with smart dresses, and with the hardly less smart gowns and hoods of the medical staff. The prize-giving took place first in the new Demonstration Theatre, Mr. Robert Gordon presenting the prizes. The rest of a thoroughly enjoyable afternoon was spent in sitting under the beautiful trees listening to the band of the 1st Life Guards, and in wandering through the wards, laboratories, &c.—in fact, where one would. Nurses in cool lilac dresses flitted about the grounds, or received the visitors with courtesy in the wards. The patients seemed to enjoy the constant stream of visitors—the cot children smiled upon the strangers, the ward tables were bright with flowers, the great hospital was en fête. It was a charming meeting-time for old friends, and if some of us felt a little like Rip van Winkle we nevertheless contrived to have a very "happy day."

### A SHAM NURSE.

It is satisfactory that one of those pests of society, a woman assuming the attire of a nurse in order to obtain better facilities for carrying on her nefarious practices, has been brought to book. The culprit was accused of stealing a brooch at Whiteley's, and when she appeared in the dock at Marylebone she wore a nurse's dress, and described herself as a member of the profession. It transpired that she was a practised thief, and had resorted to the expedient of posing as a nurse, with the idea that her respectable appearance would conceal her true calling. No doubt it served her purpose for a time but for three months, at any rate, she will not have th opportunity of masquerading in a dress to which she i not entitled. Prison costume is certainly more appropriate.

### AN ASSOCIATION OF TRAINED NURSES FOR SYDNEY.

On Friday evening, June 2nd, a large and representative meeting of doctors and nurses was held in the Royal Society's Rooms, Sydney, N.S.W., to consider the best means of forming an Association of Trained Nurses. Sydney is the happy hunting ground for impostors of all kinds, and the profession and public have suffered severely from the misrepresentations of so-called nurses for many years. A few years ago steps were taken to organise some method of dealing with the evil, but, unfortunately, it fell through. There is, however, every reason to hope that a definite organisation will be the outcome of the present movement. At the meeting it was decided to form an Association of Trained Certificated Nurses who have received at least three years' hospital training; that the mimimum number of beds for general hospital training should be 20, and for private and special hospitals 12; that those desirous of entering from private and special hospitals should do so within the first six months after the founding of the association; that all nurses be classified according to work and training; that an entrance fee of a guinea or half-a-guinea, according to grade, be imposed; that all nurses be required to produce references from the medical superintendent and matron in addition to their certificates, and that in the case of private nurses references from two doctors and two patients will be necessary; that a committee be formed consisting of 20 members, half medical men and the remainder matrons, nurses, and lay members; and that distinctive badges be worn with the name of the hospital where nurses received their training. It was then suggested that a Central Bureau should be formed to transact all business of the association; and that a Mutual Benefit Society should be formed in connection with it in the interests of sick or aged nurses. At present there is nothing of the latter kind in Sydney, and in a few years the need of it will be urgently apparent.

### BIG BEN'S BOOM.

Owing to the illness of a lady, the night chimes of Big Ben have been suppressed. The patients of the Westminster and St. Thomas's Hospitals would reap material benefit if the veteran's ding-dong were heard no more in "the stilly night." A former patient of St. Thomas's correctly affirms that this striking is a source of great suffering to the sick lying within earshot. Perhaps a petition from the authorities of the hospitals might result in alleviating the annoyance caused to the inmates by the tongue of the offender.

### A NURSES' HOME FOR BLACKBURN.

It is significant that at the third annual meeting of the Blackburn District Nursing Association Dr. Martin said "it was a thousand pities that the association was not established years ago." He then drew an effective picture of the work in which the nurses of the association are engaged, and mentioned as among its advantages the fact that the people are taught nursing, certain habits of cleanliness, how to pourtray any symptoms in a patient when they arrive, and obedience to the instructions of the medical men. On the latter point Dr. Martin contended that "it did not matter what sort of a medical man they had if his experience and his orders were set at defiance by any old 'gamp,' for the patient could not in that case benefit by all the doctor's attention." The proposal to build a home at the cost of £5,000, including the site, which was the feature of the annual report, was cordially adopted. There are now six nurses in full work, and, as a greater number will soon be required, the need of proper accommodation for them is obvious. The new home is to be on modern lines, but the plans have not yet been passed.

### SUPPLY AND DEMAND.

According to the *Eastern Morning News*, "all the chief centres are glutted with nurses of one kind or another, good, bad, and indifferent." We presume that the chief centres indicated are Hull, Grimsby, and neighbouring towns. If the statement of our contemporary is accurate, and the supply of nurses in that part of the country where it circulates is in excess of the demand, some of the ladies should move further south. There is Hampshire, for instance. The Hampshire Nursing Association, of which Lady Selborne is president, cannot secure anything like a sufficient number of nurses. Many districts are now waiting for them. So, too, at Chertsey the dearth of nurses is so great that some of the guardians are advocating the training of probationers on the spot. This is practically impossible, unless or until a resident medical officer is appointed. As an inspector of the Local Government Board stated at a recent meeting of guardians, probationer-nurses do not care to go to a place where the training is not of the very highest character. But the alleged over-supply of nurses in the north-east is, we fancy, in striking contrast to the situation generally.

### THE OFFICE OF MATRON OF THE GRIMSBY NURSING INSTITUTION.

Considerable regret seems to be felt in Grimsby at the resignation of the matron of the nursing institution, and surprise has been expressed that no account of the meeting at which it was accepted has appeared in print. Miss Radcliff is generally respected in the town, and is credited with having done most excellent work. Her retirement has excited all the more comment because the changes in the office of matron at Grimsby have been somewhat frequent. It need hardly be pointed out that circumstances which lead a nursing institution to constantly change the head of its staff must require careful consideration by the committee.

### THE QUESTION OF LEAVE.

There has been some friction at Dover lately on the question of leave. The superintendent-nurse at the Workhouse Infirmary asked for leave during the time of visiting, and it was refused to her. She accordingly complained to the Board of Guardians, but they instructed the clerk to advise her that the action of the matron was in accordance with the regulations "recently made" by the Board. The matron, therefore, cannot be blamed. But the report of the case does not state why leave was asked for, or why the regulations enforced by the matron were adopted. There is all the difference in the world between asking leave for inadequate reasons and asking for it on special grounds. Nor are new regulations necessarily justifiable, though so long as they exist they must be respected.

### THE BLAIRGOWRIE NURSING ASSOCIATION AND MISS JACKSON.

It cannot, at any rate, be said that the complaint of Miss Jackson, who did not receive her full certificate on resignation of her position as a Queen's nurse at Blairgowrie, has not received the attention it deserved at the hands of the committee of the Nursing Association. At a meeting held in the Town Hall, Blairgowrie, Miss Guthrie Wright, of Edinburgh, made a long speech, in which she warmly vindicated the course pursued by Miss Wade, the lady superintendent. She denied that the certificate to which Nurse Jackson takes exception is "worse than useless," and affirmed that it is "quite a valid certificate, and would be a help rather than a hindrance to Miss Jackson in obtaining a situation." Miss Wright instanced a case of a nurse who was dismissed from the Jubilee Nurses before her two years were finished, "who not only got another situation, but was now matron of a cottage hospital." It appears that so far back as May, 1898, Miss Wade wrote to Miss Jackson finding various faults with her, and stating that if renewed grounds of complaint arose she would be badly placed towards the institute. The proceedings at the meeting leave no room for doubt as to the fact that the Nursing Association entirely approve the action of Miss Wade, and are prepared to defend any hostile movement.

### NURSES AND FRESH AIR.

A correspondent, who says that "so many nurses when off duty spend their time either in their bedrooms or sitting-rooms," asks to be allowed to describe one or two most enjoyable ways of spending an off-duty, whether short or long. "If your hospital happens to be near a river take a boat for the season; you will find this much less expensive than hiring a boat each time you go out, especially if half-a-dozen join together. Once on the river you are away from the rush and scurry of the town, out in the glorious country; work, books, or writing can easily be managed by drawing the boat under the trees and lightly fastening it. If the off-duty is long, I strongly advise taking an 'Etna' and food with you; there is keen enjoyment in making the kettle boil in as short a time as possible, and trying to lie all round it to prevent the draught getting near. The rowing exercise uses a different set of muscles to those constantly employed in the wards, and in the heat of the summer the river is most refreshing, for there is always a breeze and no dust. A bicycle is another good means of getting quickly out of the town, and one can easily go a few miles without feeling tired, and then can either loiter about the lanes picking flowers here and there and exploring the country, or can sit and read in a field without being disturbed. I have been nursing now nearly three years, and find that change in private clothes, outdoor exercise and fresh air are the very best means of keeping well and happy."

### SHORT ITEMS.

An appeal is being made to the public on behalf of the Belgrave Hospital for Children, which, much to the regret of the Pimlico people, is about to be moved from that part of the town to Kennington. The site has been secured, and it is proposed to build a portion of the structure as soon as £15,000 of the £50,000 required is subscribed.—In aid of the London School Nurses' Aid Society, a pretty and effective display by the children of the Kindergarten and North Hackney High School and School of Housewifery was given on Saturday at the Highbury Athenæum, under the auspices of Miss James, the principal.—A bazaar and café chantant was held on Tuesday afternoon, at the house of Lady Trevor, in aid of the Victoria Hospital for Children. There was plenty of good music and singing, and a considerable sum must have been realised for the charity.—Dr Gunning, of Kensington, has given £1,250 for the endowment of a bed in the Dumfries and Galloway Infirmary. —The Prince and Princess of Wales will open the new buildings of the Alexandra Hospital for Children with Hip Disease, Queen Square, Bloomsbury, on Thursday next, at noon.

### Gynæcological Nursing.

By G. A. Hawkins-Ambler, F.R.C.S., Surgeon to the Samaritan Free Hospital for Women; Assistant Surgeon to the Stanley Hospital, Liverpool.

(*Continued from page* 194.)

### ON DOUCHING.

Patients are usually douched for (1) cleansing purposes, (2) for the application of drugs to the vagina and neck of the uterus, and (3) to relieve inflammation in the pelvis. In the former cases it is usually sufficient to use one or two pints of either sterilised water, or water in which some antiseptic or other medicament has been dissolved. Common disinfectants would be corrosive sublimate lotion (one part in 2,000 or 3,000 of water), 1 in 40 carbolic acid, Condy's Fluid, Sanitas, Jeyes' Fluid, or other antiseptic. Amongst the medicaments in common use we have sugar of lead (quarter of an ounce to one or two pints of water), decoction of poppy heads, sugar of lead with laudanum in equal proportions, sulphate of zinc, alum, and many others.

The appliances are a bed-pan, a douche-can with long tube and a vaginal attachment, a macintosh sheet, sterilised water (hot), and a towel. The bed-pan must be warmed and placed under the patient as she lies in bed. The nurse will then prepare the douche of sufficient heat (from 80 to 100 deg. Fahr.), and permit a little of it to flow through the tube (which is preferably made of toughened glass that can be boiled), until she has made sure that the tube is full, and that neither air nor cold water will enter the patient. She will now grease the vaginal piece with plain or carbolised vaseline or carbolic oil, and, after gently separating the labia, will pass the tube within the vagina for three or four inches very gently and steadily.

She will now turn on the water, which flows into the patient and out again into the bed-pan. When used for cleansing purposes, as in preparation for operations, it would be well to raise the douche-pan a few feet above the patient, and when she has inserted the nozzle of the tube to slip her finger alongside it and depress the posterior wall of the vagina, so that this may be stretched somewhat and permit the water to enter freely and wash away matter that has collected in its grooves and folds.

In the application of medicaments to the vagina for inflammatory conditions or for leucorrhœa it is not so necessary either to raise the douche-pan to a high level or to introduce the finger. The nozzle of the douche-tube blocks the passage to a certain extent, and the fluid tends to balloon it, so that the entering antiseptic or solution comes into close contact with all the parts, and remains for a longer period than in the former case.

The medicines will therefore be more thoroughly applied, and will have time to exert their influence better than if the vagina were merely washed out with them. Where the douche is given for the relief of inflammation within the pelvis, it requires to be continued for some time—at least for a quarter of an hour—and the water should be very hot. A temperature of 110 to 120 deg. Fahr. will be necessary here, and patients usually complain a great deal of the hot water. When used as hot as this water is apt to scald the skin as it escapes, and it is a wise precaution to smear the buttocks and the skin in the neighbourhood of the vagina with vaseline to protect it from the effects of the hot water. You will require quite a gallon of sterilised water for such a douche as this, and if you can obtain a bed-pan which has attached to it a tube for carrying away the water that has escaped you will prevent the bed from getting wet (though the macintosh beneath the patient will save this to a certain extent), and save yourself considerable trouble and the patient much disturbance in removing the bed-pan from time to time to empty it. All these things can be achieved without uncovering the patient or disturbing her much. In some cases, however, it is desirable to bring her to the edge of the bed in the position we have described for examination—called the Sims's position—placing a macintosh under the buttocks, and hanging this over the edge of the bed, so that it is draped in the form of a scoop or funnel, to carry the escaping water into a bucket placed by the bedside. Where a macintosh is not handy, a large sheet of brown paper, or one or two newspapers, would answer the purpose just as well. The nurse will, of course, take particular care that the douche-can itself has been well cleaned and wiped out with a carbolised towel, the tube cleansed and sterilised, that the water to be used has been well boiled, and that the vaginal pipe has not only been thoroughly washed and scrubbed under a stream of flowing water, but either boiled, or allowed to soak for some hours in an antiseptic solution. It is not an uncommon experience to find that obstetric cases do perfectly well until douching has been commenced, after which we frequently see the temperature run up, because what is used as a cleansing process becomes, owing to the carelessness of the nurse as to the cleanliness of all her appliances, a means of introducing filth.

After the douche has been administered the patient may be put back to her place in bed, and the macintosh and towel be permitted to remain under her for a little while to catch any superfluity of water that may drain away from the distended vagina. Sometimes, where the prolonged action of a medicine is required, it is well to tilt the patient's pelvis by placing a pillow under the hip or buttocks, which allows the injected solution to remain for some time in contact with the walls of the vagina.

In all cases the nurse must know what the douche is required for and the time during which it must flow before she can give it with any degree of success, and it must not be given in the rough-and-ready manner too frequently observed, but with an intelligence born of the understanding of its object. It is a wise precaution to take the temperature of the douche about to be administered with a bath thermometer before giving it. There is no more frequent cause of error than that of depending on hands that may be too tender or too case-hardened to properly appreciate the heat of the water into which they are dipped.

### St. Saviour's Homes, Hendon.

The homes at Hendon for the care of feeble-minded children were not long since put under the superintendence of Miss Anderson, who was chosen to organise the Meath Home for Epileptics, Godalming. Miss Anderson was trained at St. Bartholomew's Hospital, and afterwards night superintendent at St. Mary's, Paddington, so that she is well qualified for dealing with such work. St. Saviour's Homes are a picturesque block of buildings grouped round a grassy quadrangle, and surrounded by a garden, and looked their best last Saturday on the occasion of the annual meeting and "At Home." The boys and girls are taught many things, such as laundry work, gardening, basket making, and as much elementary reading, writing, and arithmetic as their defective mental capacity is capable of receiving. The work of classifying children of less than normal intelligence is one of the most recent birth, and has undoubtedly a beneficent influence upon the community. All information may be obtained from the Secretary of the National Association for Promoting the Welfare of the Feeble-Minded, 53, Victoria Street, S.W.

### A Year's Plague Nursing in India.

By a Sister.

### NATIVES AND TREATMENT.

How was treatment at first received? Having to deal with an ignorant, superstitious, and fanatical race, it can well be imagined that it was very difficult to carry treatment into effect; and when I mention what was the popular belief even amongst the enlightened natives, my readers will form some idea of the kind of people with whom we had to deal.

Many may remember having heard of the way in which some of the natives of Bombay treated the Queen's statue which had been erected there in Jubilee year. In order to express their displeasure at the well-merited punishment accorded to one of their Brahmin leaders, they tarred the statue so successfully that it took two years to remove the marks! Just about that time plague began to increase rapidly in Bombay, and the death-rate was enormously high. Accordingly, these poor ignorant people thought that by some extraordinary craft plague was ordered to rage in India as a punishment! Eventually, a Brahmin sculptor offered to restore the statue, which he did just two years after, and it was once again unveiled in its marble white purity. Plague then began to decrease rapidly, and the people believed that the Queen had forgiven them, and was removing this scourge from their land! Their idea of a hospital was too dreadful. They did not understand such things as infection, segregation, &c., and it is not surprising, is it, that, being fatalists, they thought if they were to die, why, Kismet!

Disinfecting search parties, of which I give an illustration, had many and grievous difficulties to encounter, and it could not have been inviting work to have to penetrate into those filthy dens, looking into every nook and corner to discover plague cases, sometimes hidden away ill, dying or dead in any spot where it was thought they could not be discovered. When a patient was found he was taken at once in an ambulance to the hospital. The rest of the people living in the house were removed to a segregation camp, provided with rooms, and kept there at the expense of Government until their houses were thoroughly disinfected. When it was possible the roofs of the houses, which were usually tiled, were removed to admit of the sun, the flooring dug up, windows knocked in, and the whole place, ceilings, floors, walls, &c., disinfected. This to the native mind was absolute desecration, and they could not understand it; and is it to be wondered at? Yet what was to be done in the face of such an awful epidemic, with a death rate of 75 per cent.? Strong measures had to be resorted to, though at the same time one could easily realise that the people strongly objected, and eventually showed their displeasure in a very unpleasant way. Many tales are told by members of search parties as to the way the natives tried to evade the discovery of plague in their houses. For instance, it is a fact that dead bodies were discovered wrapped up in bedding which had been placed innocently against the wall, which is the usual way the natives dispose of their bedclothes during the day-time; and I was also told the following very gruesome anecdote, which was said to be true. On entering a dimly-lighted inner room four men were discovered playing cards, one of them leaning with his back against the wall holding the cards in his hand. This was not a very strange sight, perhaps, but what was the amazement of the search party when the man leaning against the wall was discovered to be a corpse, and had only been placed in that position to evade the discovery of the officials, who were seen coming down the street. I am not exaggerating when I say that the natives actually supposed they were removed to the hospital only to be poisoned, and they looked upon us merely as tools in the hands of those in authority. I shall never forget the look of abject terror that sometimes crept into the eyes of the poor creatures when we used to give them their necessary medicines; indeed, it was heartrending, and it often puzzled me to think how they could possibly look upon us as such inhuman monsters! It was sometimes with the greatest difficulty that we could even induce them to take any nourishment. They would keep their mouths obstinately shut rather than take what we were giving them, firmly convinced in their own minds it was poison. It was particularly unfor-fortunate that we had to give them brandy or rum, as so many of them had never taken spirits in their lives, it being strictly against their religion and caste. I am sure that some of them would willingly have died sooner than break their life long faith. However, we had to disguise the truth as far as we were able, and try to induce them to think it was only

Disinfecting the Houses in One of the Principal Streets in the City.

medicine. In many cases, when owing to paralysis we had to resort to nasal feeding—which was, of course, absolutely necessary—they strongly objected, and their relatives considered it was merely a means of torture which was sure to result in immediate death. They were very much relieved and impressed when they found that the sufferers lived for several days kept up entirely in this way, utterly unable to swallow anything by the mouth. Hypodermic injections were also looked upon with terror, and the patients fancied that if they refused to swallow the poison it was injected into them in this manner. It is hardly credible, but, alas! such was their impression at first, and it was most pathetic to see their helpless misery. Indeed, considering that 75 per cent. of our patients died in spite of all our care and attention it is not to be wondered at that they were apt to doubt our skill, and that the hospitals were looked upon as places from which very few escaped alive! I must here refer to the sad deaths of two of our nurses from plague, which occurred during the winter, and which cast a great gloom over us all. One of them died of pneumonic plague at Poona, and it was strange that, though suffering from an acute form, she retained her consciousness to the last. Her illness was a short one—on Thursday she went for a bicycle ride in the very best of health, and on returning home complained only of feeling very warm, which was not surprising, as it was a windy afternoon and she had been riding against the breeze. Next morning she felt seedy, but got up as usual and went on duty in the camp, but feeling much worse about eleven a.m. she took her temperature, which she discovered to be 103 deg. The doctor immediately ordered her to bed, from which she never again rose, and on Sunday she had all the unmistakable symptoms of pneumonic plague with heart failure. Early on Monday morning she passed peacefully away. The other nurse who went out to India with me died at Bombay, and hers was a case of direct contagion. She had been feeding a patient, which was sometimes very difficult, and in this instance he coughed in her face, and the poison entering her eye caused inflammation. This resulted in a very acute and painful form of plague, to which she succumbed. The nurses were very much sympathised with in their loss, and the Queen graciously sent a private telegram to us expressing her sorrow. Neither of these two nurses had been inoculated, and after their deaths we received urgent letters from the Surgeon-General requesting that we should all be "done" without delay, and so most of us agreed to it; and next week I will recite my own experience.

### Appointments.

The Infirmary, East Dulwich Grove, S.E.—Miss Katharine Emelia Nisbet, second assistant matron, has been appointed First Assistant Matron at this infirmary. Miss Nisbet was trained at King's College Hospital, and the Hospital for Sick Children, Great Ormond Street. She was matron of the English Red Cross Hospital at Chalcis, Greece, during the late Græco-Turkish war, and she holds the L.O.S. diploma.

Gravesend Hospital.—Miss Marian Measures has been appointed Matron. She was trained at Guy's Hospital, and was subsequently sister at the Kent and Canterbury Hospital, and at the Children's Hospital, Manchester. Since July, 1896, Miss Measures has been home sister at the Dreadnought Seamen's Hospital, Greenwich.

Grosvenor Hospital, Vincent Square, S.W.—On June 1st, Miss F. M. Phillips was appointed Matron. She was trained at Soho and Westminster Hospitals. She has since been staff nurse at Soho; and, after receiving general training at Westminster, was appointed sister to the women and children's surgical wards.

### Memorial Tablets at St. Thomas's Hospital.

Three memorial tablets have recently been erected in St. Thomas's Hospital over beds endowed in perpetuity. One in the "Albert" ward bears the following inscription: "Tom Hughes's Bed. In memory of Tom Hughes, Q.C., author of 'Tom Brown's School Days.' Born 1823, died 1896." Another in the "Florence" ward is thus inscribed: "The Woolf Joel Bed. In memory of Woolf Joel, 1898." On the third in the "Edward" ward is engraved: "Richmond Bed. In memory of Sophia Richmond, 1899." The work has been carried out by Messrs. Cox, Sons, Buckley, and Co., of Tavistock Street, Covent Garden. The tablets are of brass surrounded by moulded red marble borders.

### Typical Patients.

### THE PLUCKY CHILD.

He was from the country, as anybody could see. A sturdy boy, with bright rosy cheeks and fine strong legs, one of which had come to grief in the football field, hence his acquaintance with a hospital. The little sufferers in the surrounding beds looked very pale and sickly, though some of them were merry little fellows, who found ways and means of amusing themselves, as little boys will, in spite of splints, cradles, extensions, and other inconvenient appliances. Our little friend, whose name was Matt, looked with grave, wondering eyes at the strange faces around him, eyes that grew a little wistful as they rested on his mother's face, the one link left to him of home; a slender link, too, which might give way and depart at any moment, leaving him in this land of strangers, if the house-surgeon should decide to take him in. The ward sister arrived, and two or three doctors. One of these was a great man, but Matt, who did not know any better, thought he was a little man. His greatness became more apparent later when it was his word that caused this or that treatment to be carried out, his consent that must be obtained before the patient might sit up in bed, or have his clothes on, and finally—go home.

They examined the wound, they touched it and pressed it just where it was sorest. Most little boys would certainly have howled; but Matt uttered never a sound. They looked at his face, but, except for a slight heightening of the rosy colour, he made no sign. "Does it hurt you, boy? Say if it hurts you." (Probe) "That hurt? What?" No answer. "Tell them if it hurts you, Matt," said his mother. (Probe again.) "Doesn't that hurt?" "No, sir," in a thick, gruff little voice. Well, they kept him in, and "mother" went away with her soft eyes very full of tears. Again the wistful look in Matt's, and a very gruff little "good-bye," but no tears, nothing of the kind. Had the boy no feelings at all? Was it pluck or blunt insensibility? The night nurse could have told. Not that he gave her any trouble, for he was as good as he had any power of being with a temperature of 103; with wide open, troubled eyes that could not close in sleep; with ears strained to explain every unwonted sound through all that long, long night. In the early morning he had a little nap. He thought he lay awake all night and watched it get light, and the slowly-returning daylight brought him a strange comforter. This was nothing less than a tall red chimney, pouring forth dense smoke into the chilly air of the grey March morning. He could see it from the window, and it filled him with a sense of comfort and of home. His face was radiant when the nurse came with basin and towel to wash him. He even grew confidential, and told her the secret of his new delight. His father worked at a coke-oven, and there was a big red chimney there with smoke like that. He saw it from the train as he came in, and father waved his handkerchief. He was quite sure that was father's chimney. What time was it, please? Father got up early and would soon be there. It was not father's chimney, and little Matt's geography was at fault; but for all the nurse knew it might have been, and had she known she would have been the last to rob him of his bit of comfort. Of course, Matt was a great favourite with doctors and nurses. Little boys who don't howl generally are. And all Matt's pluck was needed before the leg was mended. But at last the happy day came when he said good-bye to nurses, patients, and to his friend the chimney. He presently found himself at home again in the sweet country air, and in the dear white cottage by the burn; with the pear tree just budding into blossom, and the white lilac bush by the door putting forth new green leaves. "Why did you never let on, Matt, when they hurt your leg?" asked his mother, when all these trials were things of the past, and the black cat purred upon his knee. "Because," said Matt, with his quaint, stolid smile, "because I wasn't going to have them laughing at us."

### Nursing of Tracheotomy Cases.

By a Nurse.

Tracheotomy, or the operation of cutting an incision in the trachea, is performed upon children and adults, but is most common amongst the former. It is done either for the purpose of letting in air or letting foreign bodies out. The opening can be made either above or below the thyroid gland, but is most commonly made above. When the opening is made a silver tube is inserted. This tube consists of two parts, the outer and the inner. The outer one is secured in the wound and kept in position by tapes which fasten round the patient's neck, the inner one being removed from time to time for cleansing purposes.

The most common causes of obstruction to the entrance of air are:—

A. Disease.—Croup, enlarged glands, growths, diphtheria, ulceration, aneurism, &c.

B. Injury.—Swallowing hot drinks or corrosive fluids, from blows or impaction of foreign bodies, or with children it is often found that they have been drinking boiling fluid from a kettle or teapot.

The obstruction may be continuous, as by the blocking up of the natural passage by membrane or swelling, or it may be spasmodic, the glottis closing by reflex irritation. In croup there is continuous dyspnœa, aggravated at intervals by paroxysms of suffocation. Whether the patient be a child or an adult it requires the same careful and good nursing. A case of tracheotomy is one of the most anxious cases that a nurse has to attend to, especially away in a country place perhaps miles from a doctor.

There are four things which a nurse has to pay special attention to: (1) Attention to temperature of the room and steam kettle; (2) keep the tube clean; (3) feeding of patient; (4) avoid infection.

1. The room or steam tent should be kept at an even temperature from 65 deg. Fahr. to 70 deg. Fahr., as there is always the danger of pneumonia or bronchitis. Some surgeons prefer to do without the steam tent and kettle, whereas some like a kettle only. If in hospitals and these things are needed there is never any difficulty in getting them, but in private houses and schools a nurse often has to improvise both. A steam kettle is easily arranged by having an ordinary one and rolling up brown paper and fixing it on the end of the spout; the water must not come above the level of the spout. Sometimes plain water only is used to moisten the atmosphere, whereas some surgeons like it to be medicated; this is done by adding a few drops of tincture of benzoin, creosote, terebinth, or eucalyptus. A tent is also easily contrived by having four laths of wood and fastening them to each of the four corners of the bed or cot and if possible four more pieces to go across, but two would do if four could not be obtained. The whole of this, except the half of one side, is covered with sheets, care being taken to turn one corner back of the sheet which covers the top; this allows a little of the steam to escape, thus preventing the atmosphere of the tent becoming too damp.

2. Keeping the Tube Clean.—When removing the inner tube for cleaning the nurse must always place the finger and thumb of the other hand on the outer tube, pressing it gently towards the wound. There is often difficulty in getting out the inner tube, as it is so often stuck by the sticky mucus or portions of membrane; constant cleansing is required, or the patient may suffocate. When the inner tube is removed it is well to let it soak for a few minutes in an alkaline solution, such as warm lime water or bicarbonate of soda (about fifteen grains to the ounce). Whilst this is soaking the outer tube may be cleansed by means of a feather dipped in the alkaline solution as mentioned above. This cleansing process requires to be done frequently, especially during the first forty-eight hours after tracheotomy has been performed. If there is any difficulty in expelling the mucus, or the breathing does not seem satisfactory, the surgeon should be summoned. If pieces of membrane be detruded, they should be saved for the surgeon's inspection, care being taken that they are well covered with a glass cover and a piece of lint or linen soaked in $\frac{1}{20}$ carbolic placed under the glass cover. Some little knack is required in catching the membrane, as it is partly expelled, or it will be soon sucked in again. A large secretion of mucus is a good sign during the first few days. A dry tracheotomy is a dangerous one. A nurse should always have a pair of dilators at hand in case of emergency.

What is a nurse to do if the outer tube should be coughed out? In hospital she can always send for the house-surgeon, though even then the patient may die before he arrives. How much more is this likely to happen in a private house? In this anxious moment the first thing for a nurse to do is not to lose her head, but without a moment's hesitation to get the patient into a proper position; that is, if a child, take it out of its cot and place it across your knee with its head thrown back, or lay it on a table with its head thrown back (a firm roll of something is placed under its neck). If this accident happens to an adult the best thing to do is to get him to the edge of the bed and let him hang his head well over the edge. If the nurse is unable to insert the tube she can keep the wound open with a pair of dilators, forceps, or even a bent hairpin until assistance arrives.

3. Feeding the Patient.—This is of the utmost importance. The patient should be given some nourishment every two hours at least, very young children require it more often. In cases where there is much exhaustion, and the patient is unable to take a sufficient quantity by mouth, nasal feeding should be resorted to. For this purpose a soft catheter (No. 4 to 6) should be passed through the nostril on to the back of the pharynx, and so on down the œsophagus. By this means more fluid can be given at a time, so that the patient can sleep longer without being disturbed.

*Note.*—Great care must be taken (before beginning to pour the fluid into the funnel) to make sure the tube has passed down the œsophagus, and not the trachea, as if passed down the latter it would set up pneumonia and probably death would follow.

4. Avoid Infection.—This is a most important item. Be most careful to disinfect everything that has come in contact with the patient, even though it may not be used for anyone else; also be most careful to disinfect your hands. Never touch your face or eyes after touching your patient before you have thoroughly washed your hands with soap, water, and nail brush, and disinfected them; merely dipping them in perchloride, carbolic, or some other solution is not sufficient. Always watch for heart and lung complications in cases where tracheotomy has been performed. When there is bad dyspnœa it is often greatly relieved by placing a sponge over the tube which has been wrung out of boiling water.

### Death in Our Ranks.

General and heartfelt regret is felt in New South Wales at the death of Miss Mary Goulding, matron of the Grafton Hospital. Miss Goulding contracted typhoid fever from a nurse whom she attended during her illness. The deceased lady was trained at the Prince Alfred Hospital, Sydney, and carried from there high credentials when appointed head nurse at the Grafton Hospital. On the retirement of her predecessor she was unanimously elected to the vacant matronship, and held the post for three years, winning the respect and affection of all with whom she came in contact.

### Nursing Scarlet Fever Convalescents.

A CHAT WITH MISS MARY WARDELL.

The heights of Brockley, on which the Mary Wardell Convalescent Home for Scarlet Fever is situated are easily accessible from the pretty station at Stanmore. When I went to see the founder, for a chat about the home which she first thought of twenty years ago, the driver, whom I asked to put me out at Miss Wardell's private residence, deposited me in a charming shady spot, known as Wood Lane, opposite a gate with "Sulloniacæ" on the post. Not that Miss Wardell does not live on the spot. Her private residence is, in fact, a part of the same block of buildings as the institution, though quite a distinct house; and as I approached the entrance I caught a glimpse of some happy looking little convalescents in the adjoining home.

"You might tell me, please, Miss Wardell," I said, "how it was you came to think of starting the Convalescent Home for Scarlet Fever?'

"When I was working among the poor in London and had a Bible woman, I tried to send two children who had recovered from scarlet fever to a convalescent home, but nobody would receive them. Then my Bible woman had a mild attack, and I could not get her taken in anywhere afterwards. Eventually, when I had discussed the matter with a few ladies, I broached it to several eminent members of the medical profession, and one of them in encouraging me to start a home myself said, 'You will succeed, and you will be one of the great benefactors of the age.' I could not refuse after that. My first idea was to found a charitable institution only."

"But you modified your project?"

"Yes, I was advised that it would be more likely to succeed if I had paying patients, some of them belonging to the working classes and paying a portion of the cost of the institution, and others paying a moderate fee. The next step of importance was a meeting at the Mansion House on behalf of the Mary Wardell Home, and five years later, on July 14th, 1884, this building was opened by the Princess of Wales, the patroness. The great feature is that it is the only home that receives persons recovering from scarlet fever except the Metropolitan Asylums Board hospitals."

"Was there any house on the spot where you selected the site?"

"One partly built. It was the beautiful and breezy situation that attracted me. This wing, which I occupy, I erected at my own expense, and I intend it to be the male patients' department when I die."

"What is your present financial position?"

"There is a debt on the Home, and I am just now endeavouring to raise £2,000. That sum would also enable us to pay for the cleaning and painting done last year, as well as to effect repairs much needed, and to replace property destroyed by a recent fire. So far, in two years, I have only got £700 towards the amount. Since that appeal was made the question of drainage has arisen. When the home was built no public sewer was within reach of the property, and it was found necessary to provide a special system of subsoil irrigation for the disposal of the drainage. Now the District Council have constructed a public sewer, and require us to lay down a completely new drainage system, to communicate with their main drain. This means that we want another thousand pounds."

"That is to say, £3,000 in all would free you from all liabilities and put the place on a sound financial footing?"

"Exactly. I should then be able to save the payment of interest on money advanced by the bank."

"What do you consider the yearly cost of the home?"

"About £2,000. The patients pay about £1,000 and our subscriptions and donations amount to about £600. Another £300 or £400 would ensure both ends meeting."

"I should like," continued Miss Wardell, pathetically, "to see the home put on a sound footing before I leave."

"Do you remember how many patients you have had since it was opened?"

"Upwards of 3,150. I often have applications for male patients in the second-class ward, and it cuts me to the heart to have to refuse to admit them."

"How do you divide the home?"

"The first floor is reserved for bedrooms first class, and the second floor for second class patients, the ground floor containing the sitting-room. The home was built to accommodate forty inmates, but we have had as many as forty-seven. To show you how the number varies, I may mention that on one occasion we only had one patient, and the very next week I had so many applications that I could not entertain them all."

"I suppose you have to keep a large staff?"

"Yes, all the time. In addition to the matron, assistant matron, and sister, there is a cook, kitchenmaid, parlourmaid, two housemaids, and a scullerymaid. The matron is a lady housekeeper, and the assistant makes the linen and gives out the stores, &c. The sister, who takes the head of the second-class table, is, of course, a trained nurse. We have also a sort of probationer, who attends to the little children and waits upon them."

"Do you often have sad cases of little children?"

"Frequently. One little boy who used to lie by the fire on a pillow was so emaciated at first that it was delightful to see him run after a time. He was the son of a dock labourer, and I did not like sending him home. We had a little girl from Shadwell who had hip disease, and she cried when she had to go."

"Now, to deal with the treatment, Miss Wardell? How do you begin?"

"We send the omnibus for the patient. It is a private omnibus, and patients travelling here by any other conveyance without special permission are refused admittance. Patients are admitted only on the subsidence of acute symptoms of scarlet fever. We frequently put the omnibus on the rail. I have sent as far as Staffordshire."

"What happens to the patient on arrival?"

"The first-class go to their room. The treatment depends upon the kind of case. The morning after arrival a patient has breakfast in bed. Then the sister makes a proper examination, and if there are traces of albuminuria the patient is kept in bed or on a milk diet until the doctor comes. Each case is carefully watched. Sometimes we have patients who do not obey the rules. There was a gentleman who would go outdoors, caught a chill, and set up albuminuria, with the result that he never got rid of it. Any tendency to rheumatism is at once observed, and the moment patients show signs of tonsilitis they are put apart."

"How often does the doctor attend?"

"Twice a week, when he sees any of the patients. He also comes whenever he is wanted, but in such cases extra is charged. No patient is allowed to leave the home without the doctor's certificate."

"Do you often get cases that want much watching?"

"No, only now and then. The mild cases of fever are long peeling, and more prone to develop rheumatism than the cases that have been acute."

"What about diet?"

"The first-class patients have anything they require, as in an ordinary home. The second-class have porridge, &c., for breakfast at half-past eight, milk and biscuits at eleven, dinner at one, tea at five, and supper at eight. Children over twelve sit up to supper. A special diet is provided in cases of illness.

"Does the sister stay long at the home?"

"Usually not more than a year or two. The sisters are very happy and like their work, but they have to give up their surgical practice, and, as one of them said to me, 'If I stay here I lose it all, after having paid for my knowledge.' It is one of my great troubles that a sister seldom remains for more than two years. A nurse came from the Nightingale Home as a patient, and afterwards stayed here as sister for two and a-half years. I may say that there is a great deal of important work in testing and watching symptoms, dispensing and disinfecting."

And then, having strolled round the lovely grounds with Miss Wardell, and seen the laundries and disinfecting apparatus, as well as the two oaks presented respectively by Prince Christian in 1887, and the Queen in 1897, I left the pleasant home without a doctor's certificate.

### A Book and its Story.

### THE SOLITARY SUMMER.

Out from the throng and the stress of the hurrying crowd, away from whatever at this moment irks and frets the spirit in the performance of uncongenial duties, whether social or professional, I ask my readers to come with me into the cool calm of that summer retreat from whence "Elizabeth" has sent forth her chronicle of the months from May to September.* In a previous book it is explained to her readers that she is the wife of a German husband, but an Englishwoman herself. Like all her countrywomen she loves a garden very dearly. In a review of "Elizabeth and her German Garden" a leading contemporary found the authoress "a little fantastic and wayward here and there." To us this little touch of phantasy and waywardness, among much that is deep and poetical, is the peculiar charm of her writing. There is, too, such an atmosphere of peace about the chronicle of these quiet days of retirement that we feel sure the book will be very welcome to those who perforce live far from scenes like those described with such picturesque fidelity.

Elizabeth's husband, inscribed "The Man of Wrath" and "The Sage," smiles indulgently at her vagaries. She suggests that she is misunderstood, but it is evident to the onlooker that he receives her proposals with gravity and without the effusion she would naturally desire, because he regards all feminine projects as best left to their own development and to those elements of destruction found within themselves.

Her one desire is to get away into the depths of the country, freed from the banalities of German provinciality and its bourgeois environment. On the plains and in the forests then, there at last she will find silence, and "where there is silence I have found peace." To the expression of this longing to be face to face with Nature the sage replies briefly, "Mind you do not get your feet wet."

Elizabeth has an unfailing source of solace and amusement in her three babies, "April," "May," and "June," surely the mother's very own, with their quaint whimsicalities. The cat "Moses," too, is an important personage much beloved of them.

"Do you love Moses, mummy?" asked the May baby, jumping into my lap and taking my face in both her hands—one of the many pretty, caressing little ways of a very pretty, caressing little creature.

"Yes," I replied bravely, "I love him."

"Then I, too," they cried with simultaneous gladness, the seal having thus been affixed to the legitimacy of their regard for him.

In order to facilitate the acquisition of another tongue, Elizabeth engages a French governess for the children, who, finding they cannot understand her, promptly settle the difficulty by teaching her German. "Poor little Séraphine." "They are three to one, and very zealous, and she is a meek little person with a profile like a teapot, with a twisted black handle of hair." Under their direction she acquires a rapid knowledge of her mistakes, at any rate. In time they possess a mixed vocabulary, in which they apply occasionally those French words which have struck them as worthy of notice. Séraphine has told them a story of a little girl who was good, and a boy who was decidedly bad. The girl having taken the right way, which led to "Himmel," has her reward. "But the boy? Oh, he was naughty and went the other way, where there is a tree, and on the tree is written, 'Don't go this way, or you'll be dead.' And he said, 'That is one *bêtise*,' and did go in, and got to the *Hölle*, and there he gets whippings when he doesn't make what the *diable* says. And, adds June conclusively, 'All boys are naughty, and I don't love them.'" Apparently no language is so sweet to the little ones as "mother's own," for they decide that "Proper mummies never speak French—only Séraphines."

In seeking retirement Elizabeth's object was "to be idle, to have time for her soul to grow, to get to the very dregs of life." And in passages of unusual beauty, when her heart goes out into the very soul of the animate life around her, drawing it in, mingling her own impressions with its vitality and loveliness, the reader who is sympathetic will rise with her into this spiritual region, and there, we hope, find for a happy space rest and satisfaction. To be dead to the world for a time, to go where no visitors can reach her, "to spend months in the garden, on the plain, and in the forests; on wet days, to go into the deepest recesses, where the pine needles are everlastingly dry; and when the sun shines, to lie on the heath and see how the broom flares against the clouds, will be perpetual happiness, because there will be no one to worry me." But German gardeners, French governesses, and other members of her *ménage* contrive to make domestic "occasions" sometimes for her.

Here is an extract full of charm, bringing the simple country life vividly before us: "It is clear that I was born for a placid country life, and placid it certainly is; so much so that the days are sometimes far more like a dream than anything real, the quiet days of reading and thinking, and watching the changing lights, and the growth and fading of the flowers; the fresh, quiet days, when life is so full of zest that you cannot stop yourself from singing because you are so happy; the warm, quiet days, lying on the grass in a secluded corner, observing the procession of the clouds. Each morning the simple act of opening my bedroom windows is the means of giving me an ever-recurring pleasure. . . . A century ago a man lived here who loved his garden. He loved, however, in his younger days travelling as well, but in that little corner of the earth belonging to him he brought back the seeds of many strange trees, such as had never been seen in these parts before." And it is to this past-day owner that Elizabeth pays her tribute of gratitude, especially for the grove of white, violet, mauve, and pink lilac which had survived, and scented the air with its delicate fragrance. "It grows here as nothing else will and keeps his memory, in my heart at least, for ever gratefully green."

One of the attractions of "The Solitary Summer" is the form in which it is put together. When the mood was on Elizabeth to write she, apparently out of the fulness of an impressionable poetic and yet withal not unpractical mind, set down in her diary at some length the humorous, pathetic, or philosophical ideas by which she was moved. Their record is one which we, and many other readers, will, we know, most gladly welcome and gratefully acknowledge.

* "The Solitary Summer." By the Author of "Elizabeth and her German Garden." (Macmillan, publisher, London. 6s.)

### Echoes from the Outside World.

### AN OPEN LETTER TO A HOSPITAL NURSE.

THE third little daughter of the Czar of Russia, who is called Maria, was christened at the beginning of the week, and according to a private letter from St. Petersburg, the distress of the Imperial couple at the new baby being another girl has not been one whit exaggerated. Never was a poor little girl less welcome, I fear, to the Russian people, though the Czarina, with her loving mother's heart, will probably cling all the more to the wee blossom who was not wanted. This fresh disappointment has been further accentuated by the death of Prince George, who has at length succumbed to consumption, the malady from which he has suffered for so many years. His condition had become so critical that, had he lived, he would have voluntarily renounced his right of succession in favour of the Grand Duke Michael, now the only remaining brother of the Czar. The latter, who becomes Heir Apparent, is 21, is a bright, clever young fellow, and said to have been the favourite son of his father, the late Emperor Alexander III.

IF any of your relatives are volunteers, and very few of us have not a father, a brother, or a cousin in the force, you must have been a good deal interested in the great review on Saturday. Notwithstanding the tremendous heat, I decided that I must go and see the march past, and for an hour and three-quarters we watched the steady moving of nearly 27,000 men. The bands and the cheering of the multitude sent up my enthusiasm to the highest point, and I felt as if I should like to shout as loudly as the boys around me. I was quite rejoiced when I discovered a party of Americans near by, because really our men looked extremely well. The fatigue and the weather had no appreciable effect upon them; they marched as firmly and held themselves as erect as if they had just turned out after a night's rest, and it was delightful to me to hear the complimentary remarks made on them in the quaint American style. As I came home, almost too worn-out to stand, I found the dominant feeling created by the afternoon's display was a sense of security such as I had never realised before. With so many thousands of men such as these to guard our shores, even the fear of invasion loses much of its terror.

HESSE may be only a small state, but it is decidedly a progressive one. The latest Act passed by the Diet imposes a tax upon unmarried men. Bachelors are to pay 25 per cent. more rates than men who have given hostages to fortune. Of course, the idea is not new, because if I remember rightly the Romans granted certain exemption from taxation to the father of three children. But though the possibility of such a thing in England has often been discussed, there has never yet been a member of Parliament who has dared to propose it seriously. On the other hand, I have often heard girls argue for it quite hotly. The notion of a great many would-be matrons seems to be that directly a young man finds he is paying more taxes than he would have to pay if he were a Benedict, he will at once fly to matrimony to redress his wrongs. I am afraid that such an argument scarcely shows a logical head, for surely the expenses of an establishment to keep up, with wife, children, and servants to provide for, must take a great deal more cash than the added 25 per cent. per annum. In truth, all the taxation in the world will never make men get married who don't want to. As far as my experience goes, there is only one event which makes matrimony the desire of a man's life—and that is, meeting the right woman!

THE greatest novelty at garden parties this season is an exhibition of Wireless Telegraphy. Of course a certain amount of space is necessary, but then the people who give garden parties in a fair-sized back yard are, fortunately for the guests, few and far between. At a garden party to which I was invited last week the batteries, &c., were fixed in a summer-house, and messages were transmitted to a tent erected at the farther end of the lawn. The gentleman who had put up the apparatus was in attendance to explain the why and the wherefore of everything, and the only complaint I had to make was that such was the thirst for knowledge, that the operator was kept quite busy answering questions, and was therefore only able to transmit messages intermittently. The funniest part of the entertainment was the amount of second-hand information which was flying about. So many of the questioners, having received a little instruction from headquarters, felt called upon to hand on the facts they had acquired to any other visitor standing near, and I must say that the explanation did not always increase in lucidity as it passed along.

ACCORDING to a well-known New York woman who is now over here, the most objectionable trait in the Englishwoman is her patronising manner. This stranger within our gates says that the whole time we are talking we never seem to forget that we are the old country and America is the new; and treat all the Americans as if they were nice, clever children, to be encouraged and made much of. I have not had very much to do with American girls, but I am sure I should never personally feel inclined to patronise them, for I usually find myself lost in admiration at their superior smartness and originality. The same lady says that, though the Englishwoman is very careful about the floors being scrubbed, she is not nearly as particular about the overhauling of cupboards, the frequent changing of bed linen, and the inspection of all sanitary arrangements as her American sister; while another point in which we are deficient is in the use of vegetables in our cookery. Potatos, cabbages, and cauliflowers are all we have to fall back upon during the winter because we do not understand how to cook tomatoes and "the delights of corn have not entered our imagination." Moreover, we do not sufficiently appreciate cranberries. I am telling you all this because they say a knowledge of one's faults is half way to amendment, and I daresay there is something in her strictures. But it is nice to read that in all districts in England the critic has found "an amazing preponderance of happy homes, husbands and wives living in perfect accord with bright children, whose cheerful lives are but a reflex of their parents' way."

HAVE you red hair? Because if so, instead of remembering the many times when, as a child, you cried because you were called "carrots" or "ginger," you may just pat yourself on the back and hold up your head very high, because you are in the front van of the fashion! The hairdressers, or, rather, the artistes in hair—the name by which, I believe, they prefer to be called—are continually being asked to give everyday "brown" or despised "straw" coloured hair a lovely reddish hue, and numerous are the bottles of dye which find their way on to the heads of fashionable folk. The best of the fashion, as far as the "artistes" are concerned, is that naturally the dye wears off unless regularly applied, so that a hair-dyed customer has to pay continual visits for the renewal of her beauty. Those women who are wiser (or shall I say less foolish?) wear a semi-wig over their own hair, and thus effect a "transformation," which, from the view of cleanliness and comfort, must be preferable to dyeing it, though an abundant crop of one's own hair, covered by a red wig of someone else's, must be rather warm for the wearers these hot days. A further demonstration of the adage, *Il faut souffrir pour être belle.*

### Invalid Furniture.

A visit to Messrs. Leveson and Co.'s extensive premises (90 and 92, New Oxford Street) is a pleasure as well as an education. Every form of appliance in the way of furniture that the ingenuity of man can devise for the relief of his suffering fellows has been brought to the highest point of perfection, and at the most reasonable cost. It is a great advantage that every article on view is kept in stock, so that all delay is obviated and goods are delivered on receipt of an order, carriage paid, to any part of the United Kingdom. An exhaustive catalogue, which is supplied gratis, affords to intending purchasers at a distance all necessary information, and the profuse illustrations with which its pages are adorned convey to the mind more graphically than any description the nature and design of each article. Perhaps one of the most delightful novelties is Leveson's improved cane lounge, of which we give an illustration. It is strongly made but extremely pliable, and easily adjusted to any angle of the body. It is provided with a reading desk, and complete with cretonne cushions, stuffed horsehair, costs £3 12s. The "carrying chair," of which also we give an illustration, is another useful novelty. By releasing a small catch or bolt it instantly folds quite flat and will go under the seat of a railway carriage. It is very light and strong enough to bear the heaviest weight. There is also a cheaper variety in cane, with bamboo poles, equally durable and strong, the price of which is 25s. The Merlin chairs are admirable both in shape and finish, and they abound in every variety, from the plain polished birch frame to the most elaborately stuffed and upholstered design. Reclining chairs are another luxurious novelty, and may be converted into the most comfortable of couches according to the fancy of the occupants. Of Bath chairs, spinal carriages, ambulances, and stretchers there is an enormous choice. The wicker bath chair with hood and apron (see illustration) is, perhaps, one of the newest designs. It is mounted on steel springs and has strong bicycle wheels with india-rubber tyres, whereby all irregularities of locomotion are obviated. A self-guiding front wheel and handle is another advantage, and the price complete of this wonderful vehicle is only £12 6s. For a guinea extra it may be had with Cee springs. It only remains for purchasers to send for a catalogue, and we can promise them that they will be in no way disappointed with the result of their investigations.

### Fete on Behalf of the Hammersmith Nurses.

On Tuesday afternoon the Princess Louise, accompanied by the Marquis of Lorne, and attended by Miss Paget and Colonel Collins, visited Hammersmith in order to attend a garden fête and receive purses on behalf of the Hammersmith and Fulham District Nursing Association. Her Royal Highness, who was dressed in dark blue trimmed with cream-coloured lace, arrived at Carnforth Lodge—the Nurses' Home—shortly after four, and immediately proceeded to the marquee which had been lent and erected by the County Council, to witness in the garden of the home a pastoral enacted by children, under the direction of Mrs. Lidington. The small actors and actresses were dressed as fairies, gipsies, and flowers, and were ruled by a May Queen. They performed their parts with grace and precision, and concluded the revels by singing the National Anthem.

The Royal party then took their places on the platform, and a troop of white-robed maidens, with two or three sturdy little fellows, went up to the Princess and gave her purses. Eleven different parishes sent their offerings to the association in this graceful fashion, and several ladies and gentlemen contributed in the same mode.

Major-General Goldsworthy, M.P., then delivered a short address of welcome, to which the Marquis of Lorne responded on behalf of his wife. He said he hoped that their visit would be productive of much good. District-nursing was an absolutely necessary work, but it was an uphill task to procure money for it. He trusted that the amount raised that day would defray any outstanding liabilities, and that the residents would come forward and make the financial position of the association absolutely sound. He thanked the May revellers for their entertainment. In France during the last century ladies and gentlemen played at being shepherds and shepherdesses, leading idle lives and doing no work. The shepherds and shepherdesses that hot afternoon had worked very hard in a good cause. The Royal party went round the stalls before taking their leave.

Earlier in the afternoon the annual report had been read and adopted. It shows that upwards of a thousand cases had been attended at a cost of £724. The amount received in the purses on Tuesday exceeded £95, a substantial addition to the resources of the association.

### Suicide of a Nurse in a Railway Carriage.

On the arrival of the 12.25 noon train from Scarborough at Malton on Friday a porter found a lady in a third-class carriage seriously ill. Her dress indicated that she was a nurse, and it was speedily found that she was suffering from poisoning by carbolic acid. Dr. Conacher, of Malton, promptly attended the lady, but he saw at once that the case was likely to end fatally, and within one hour of the arrival of the train at Malton she was dead. Steps were at once taken to discover her identity, and it was ascertained that she was Miss Frances Edwards, a professional nurse, who had been engaged at a nurses' home in Scarborough.

At the inquest on Saturday the principal of the home, Miss Penty, gave evidence to the following effect. She said she kept a nursing home at that place, and had twenty nurses under her. The deceased, Frances Edwards, who was twenty-nine years of age, went to her on April 5th last from Wadsley, near Sheffield. She stated that her home was in Australia—her father now lived there. She remained with witness until Friday morning—the day of her death. Witness had found out that she had been dishonest—had stolen some money—and witness told her she would either have to leave or take the place of assistant nurse, as she could not send her to private families if she were not honest. Deceased said she would leave at once. She seemed much put out, and when witness asked her where she would go, she replied, "To her friends at Sheffield." She went away to catch the 12.25 train to York, taking with her only a part of her belongings. She did not make any threats of suicide, but she asked witness if "they" had told her that she (the deceased) had left Huddersfield because she had there attempted to commit suicide. Witness answered in the negative.

Ellen Hutchinson, of Hoxton Road, Scarborough, who travelled in the train with deceased, said that when they had got about three stations away from Scarborough she saw deceased taking what she thought was some medicine from a glass. About five minutes afterwards deceased fell on the floor, but never spoke. Witness lifted her on the seat and pulled the communication-cord, but got no response to that. She then propped deceased up till they got to Malton.

Dr. Conacher said death was due to carbolic acid poisoning. She had taken a large dose.

Superintendent Jackson, of Norton, submitted various letters and telegrams resulting from inquiries he had made. Before coming to Scarborough deceased had been employed at the South-West Yorkshire Asylum at Wadsley, near Sheffield, and before that at Huddersfield, and the matron of a nursing institution there stated that deceased had on one occasion attempted to commit suicide there. She had no friends in England.

The jury at once returned a verdict of "Suicide whilst of unsound mind."

Arrangements had been made to inter the body at the expense of the parish authorities at Norton, but the matron of the South-West Yorkshire Asylum sent instructions to bury her decently at the cost of the Wadsley Institution. The funeral took place in the Norton Cemetery on Sunday morning, and the remains were followed to the grave by Miss Penty, and some of the nurses living in Malton and Norton. Subsequently, in consequence of the publicity given to the sad event, a distant relative of the deceased, who could not reach Malton in time for the funeral, telegraphed that he would defray the expense of the funeral.

### Wants and Workers.

Will any reader kindly spare some old linen for poor patients with bad legs? Address "Nurse Edith," 12, Vale Road, Hermitage Road, Finsbury Park, N.

### Minor Appointments.

Homœopathic Hospital and Private Nursing Home, Lansdown Grove, Bath.—Miss E. Thompson has been appointed Matron. She was trained at the Royal Infirmary, Preston, and has subsequently been charge nurse in the Royal Infirmary, Bristol; night superintendent at the Paddington Infirmary, London; night sister at the Cardiff Infirmary; and assistant matron at the hospital of which she now becomes matron.

Royal Albert Hospital, Devonport.—On July 1st Miss Annie Dailley was appointed Sister in the adult male ward. She was trained at the Royal Albert Hospital, Devonport. Her appointments since have been superintendent of the Ford Union Infirmary, Devonport, and sister of Monsall Fever Hospital, Manchester.

British Nursing Institute, Pernambuco, Brazil.—Miss Helena R. Butler has been appointed Nurse. She was trained at St. Bartholomew's Hospital, and has since been night superintendent and charge nurse at Johannesburg Hospital.

Indian Army Nursing Service.—Miss Helen M. Baird has been appointed Nursing Sister. She was trained at St. George's Hospital, London; and obtained her L.O.S. training under the District Nursing Association, Llanelly, South Wales.

The Hospital of St. Cross, Rugby.—Miss Blanche Sayle has been appointed Surgical Staff Nurse. Miss Sayle was trained at the Royal Infirmary, Manchester, and afterwards held the post of sister at the Hospital for Incurables, near Bury.

Royal County and City of Perth Infirmary.—Miss Rennie has been appointed Dispenser. She was trained at the General Hospital, Birmingham.

### Novelties for Nurses.

### UNBREAKABLE STEELS.

The Knitted Corset and Clothing Company, of 118, Mansfield Road, Nottingham, send us specimens of their invention, the Hercules Unbreakable Corset Steels, which are intended to supersede the usual steel and whalebone. The advantage they claim for their speciality is warranted. The steels are formed of coils of steel wire flattened, and are extremely flexible, which quality may recommend them to those engaged in active occupation. They are certainly heavier than the ordinary steel, but as they neither rust nor break this need not be an insuperable objection. The price is only 9d. for busks, and 6d. for side steels.

### Presentations.

Miss Ellen Bracewell, on leaving St. Saviour's Infirmary, East Dulwich Grove, to take up the duties of her new appointment as matron to the Mill Road Infirmary, Liverpool, was presented by the Infirmary staff with a silver tea kettle and stand, a tea tray, and a travelling clock. Miss Bracewell has held the post of first assistant matron at the infirmary for three years, and her departure is much regretted by all.

### Everybody's Opinion.

[Correspondence on all subjects is invited, but we cannot in any way be responsible for the opinions expressed by our correspondents. No communication can be entertained if the name and address of the correspondent is not given, as a guarantee of good faith but not necessarily for publication, or unless one side of the paper only is written on.]

### NURSES' READING SOCIETY.

In answer to "A Constant Reader," Miss Moberly wishes to say that she hopes to set a book on surgery for the next quarter, and she certainly proposes to deal largely with the question of modern aseptic surgery. The suggestion of a reading society has met with a most gratifying and enthusiastic response from a number of nurses, and Miss Moberly anticipates that before the next quarter begins, on October 1st, she may have been able to arrange for a lending library. Miss Moberly has been unable to answer several letters and post cards from nurses because no stamped envelope was enclosed.

### FEES IN NORTHERN NURSING INSTITUTIONS.

"Lady Superintendent" writes: May I, through your valuable paper, express my opinion on the following subject. It has often puzzled me to know why the nurses' institutions in London and the South demand (and can easily obtain) one guinea and a-half per week for ordinary cases, when the Yorkshire institutions and those of the northern counties only charge one guinea. I think the latter fee most inadequate. The nurses who work in the north are quite as capable as those who work in the south, and they, of course, expect salaries equally good. I think they would derive more benefit from their earnings if the superintendent of each institution could induce her committee to raise the fee to at least 25s. per week. Surely it is not too much to ask when women who just "take up nursing" without any training, demand, and can often obtain, one guinea per week. Moreover, the north country people are quite as well able to pay a higher fee as those in the south.

### CHEYNE-STOKES RESPIRATION.

"A Male Nurse" writes: It is very interesting to read of cases of Cheyne-Stokes respiration lasting for over three months. The majority of such cases are, I believe, to be found in our asylums. I once had experience of a case of Cheyne-Stokes respiration lasting nearly four months. The patient was a gentleman of eighty-two years of age; he had a very strong constitution, and suffered with senile dementia. Cheyne-Stokes respiration lasted nearly four months previous to death. But it is very interesting to note that the respiration was quite normal while the patient was awake, and that Cheyne-Stokes respiration set in as soon as he fell asleep. I have also met with a case of Cheyne-Stokes respiration in private nursing, but it only lasted for a fortnight. A gentleman of seventy-three years of age had an attack of apoplexy; asthenia, followed by Cheyne-Stokes respiration, set in at the end of the seventh week, and death in a fortnight later.

### CATERPILLAR RASH.

"A Nurse-Matron" writes: Will any of your correspondent give me their experience or information concerning "caterpillar rash." I am nurse-matron in a boys' school, and at present a rage prevails for collecting what are known as "bugs," which term includes insects of all kinds, both winged and creeping. Several of the collectors suffer from sore eyes, which continue bad for about a week, in spite of bathing with boracic lotion; others have local rashes of rather large spots about their faces, necks, or chests. One little boy had the whole upper part of his body covered with a small bright red rash, which became confluent and formed patches, closely resembling German measles, the only difference being that the rash was rather more raised and lasted much longer. All the sufferers are known to have handled "woolly bears" or other hairy caterpillars. A lady living in this neighbourhood got a rash very like the little boys. She had found a dead caterpillar on her the day before. The rash causes extreme irritation, but the temperature was not raised in any case. At this time last year a great many of the boys had sore eyes, but rashes were not so numerous nor pronounced. We attributed it then to the handling of caterpillars.

### TALKING SHOP.

"An Old Guy's Nurse" writes: The letters in The Hospital on the subject of "Talking Shop" bring to my mind another serious accusation which on many sides is brought against nurses. And, alas! I am not only dealing with hearsay, for I can myself bear witness in more than one instance to the truth of the accusation. Nurses are accused, too truly, of being inveterate gossips about their cases, and about the people in whose houses they are doing private nursing. It strikes me—first as a nurse, secondly as a woman with some regard for honour—that this tendency to gossip is a real disgrace to anyone calling herself a nurse. She disgraces the profession of which she is a member, and she disgraces her womanhood. It shocks me to hear, as I frequently do, nurses talking of the patients they are nursing by name, giving minute descriptions of the illness from which the patient is suffering, and, furthermore, talking volubly of the affairs of the patient's house, and of its manners, customs, and inhabitants. To my mind it is a terrible breach of honour to chatter to outsiders of the household in which you are working professionally. You have no right to tell others anything about the affairs of the people with whom you are—it is disloyal and unfair to them. Think for an instant how you would like to have your private business chattered of outside the walls of your own house. No nurse is justified in speaking of her patients by name to others. A patient is naturally averse to having his ailments generally known and made a subject of discussion; and as, owing to the nature of her work, a nurse is taken into the very heart of the family, she ought to be most particularly careful to lock up all she hears and knows of the family in her own breast. Chattering in this irresponsible fashion of the affairs of others is a dreadful breach of professional etiquette as well as a flinging away of all sense of honour.

### THE ADMISSION AGE OF PROBATIONERS.

"Ecilla" writes: At the nursing conference of the International Congress, it was suggested that probationers should be admitted younger than is now usual. It would be interesting to know the opinion of some matrons and sisters on the subject. I think no girl should nurse adults before she is twenty-three years of age nor be a midwife before twenty-five for the sake of the patient's feelings as well as for her own welfare. Very young probationers, however clever as nurses, are apt to deteriorate as women, losing their sense of delicacy of mind and speech. Physically also a very young girl is rarely fitted to bear the combined enormous mental and physical strain of a nursing probationer's life. For girls who are obliged to earn their bread from an early age and are desirous to join our noble profession it might be advantageous to take up cooking, house or nursemaid's work which would teach them discipline, method, and useful details until they are old enough to enter a big hospital. So many girls ask me what they should do and study to prepare themselves for nursing probationers, and I think the R.B.N.A. council or matrons of nurse-training schools would do good if they drew up a schedule of work and books advisable for intending nurses to practise and study. The following is the advice I usually give, adding names of suitable books. Do all you can by healthy living and exercise to perfect your own physique. Have your teeth in good order. Practise in every way to become ambidexterous. Learn sick cookery, cutting out, and rapid tacking and sewing with both hands, handle and examine parts of animals in the kitchen or butcher's shop. Study Miss Nightingale's book on nursing, and anatomy and physiology, illustrated list of instruments, dictionary of medical terms, pharmacy, Latin, and materia medica, or if possible study at a school of pharmacy for three months or longer. During my career I have been much struck with the ignorance about drugs shown by sisters otherwise highly trained, and for my patients' sakes am glad I studied pharmacy prior to entering hospital.

### For Reading to the Sick.

PEACE.

**Verses.**

Now—the sowing and the weeping,
Working hard and wailing long;
Afterward—the golden reaping,
Harvest home and grateful song.

Now—the long and toilsome duty,
Stone by stone to come and bring;
Afterward—the perfect beauty
Of the Palace of the King.

Now—the tuning and the tension,
Wailing minors, discord strong;
Afterward—the grand ascension
Of the alleluia song. —*F. R. H.*

Were half the power that fills the world with terror,
Were half the wealth bestowed on camps and courts,
Given to redeem the human soul from error,
There were no need of arsenals and forts.

The warrior's name would be a name abhorred,
And every nation that should lift again
Its hand against a brother, on its forehead
Would wear for evermore the curse of Cain!

Down the dark future, through long generations,
The echoing sounds grow fainter, and then cease;
And like a bell with solemn sweet vibrations,
I hear once more the voice of Christ say "Peace."

Peace, and no longer from its brazen portals
The blast of wars great organ shakes the skies!
But, beautiful as songs of the immortals,
The holy melodies of love arise. —*Longfellow.*

**Reading.**

TRIBULATION, PEACE, AND VICTORY.

"These things I have spoken unto you, that in Me ye might have peace. In the world ye shall have tribulation: but be of good cheer; I have overcome the world."—*John* xvi. 33.

Here are four special points—the peace, the tribulation, the victory, the cheer. It is Christ himself who is the speaker of these words. He speaks them to us—let us listen. Peace is the great Bible subject; the burden of God's message to men. Peace on earth, peace with God, the peace of conscious reconciliation. But it is not so much "peace with God" that is here referred to as "the peace of God;" not the peace obtained by receiving the embassy of peace, the reconciliation, but the peace of the reconciled soul. Into this region of peace reconciliation is the entrance. Here no wrath can reach us, no storm can ruffle us, no terror can appal us; we are "kept in perfect peace." "The peace of God rules in our hearts," and is perpetual sunshine, like an island of bright verdure in the midst of a stormy sea. It is "peace in Christ," not out of Him, nor apart from Him, but in Him. It flows out of Him to us; or rather we are in Him, and so get that peace. We get it by means of His words: "These things have I spoken unto you, that in Me ye might have peace." His words are the words of peace. The soul that listens to these words drinks in the peace, or, we may say, breathes the air of peace. Look at his words, "Let not your heart be troubled;" "In my Father's house are many mansions;" "The Father coming in and abiding;" the love of the Father; the little while; the coming joy. Yes, every word is loaded with peace; His own peace; the Father's peace; the Spirit's peace; the peace of heaven; peace even here on earth, where all is trouble and disquiet.

### Where to Go.

DOWDESWELL GALLERIES.—An interesting exhibition of Canadian and English scenery, depicted by Mr. Homer Watson in oil colour, will be on view at the Dowdeswell Galleries, 160, New Bond Street, on and after Saturday next.

### Notes and Queries.

**The contents of the Editor's Letter-box have now reached such unwieldy proportions that it has become necessary to establish a hard and fast rule regarding Answers to Correspondents. In future, all questions requiring replies will continue to be answered in this column without any fee. If an answer is required by letter, a fee of half-a-crown must be enclosed with the note containing the enquiry. We are always pleased to help our numerous correspondents to the fullest extent, and we can trust them to sympathise in the overwhelming amount of writing which makes the new rules a necessity.**

**Every communication must be accompanied by the writer's name and address, otherwise it will receive no attention.**

*L.O.S.*

(131) Could you kindly tell me of an institute where a nurse with six years' nursing experience could get free training in monthly nursing?—*L. S.*

If you advertise you may possibly hear from some of the smaller nursing homes where you can be prepared for the L.O.S. certificate in return for work. It is really cheaper in the long run to go into a hospital. See query "Membership."

*Membership.*

(132) Will you be kind enough to reply to the following? Having had only six months' training at a children's hospital, is it possible for me to belong to the Royal British Nurses' Association or be registered as a trained nurse? I left owing to my health giving way from diphtheria, followed by rheumatic fever. I have since been nursing privately, first at institutions and now on my own account. 2. Can I obtain the L.O.S. diploma whilst I am still at work? What means must I take to do so, and about what would be the probable expense?—*Cecilia.*

As training in a children's hospital counts for nothing in the nursing world, and as the Royal British Nurses' Association requires a three years' hospital certificate before admitting candidates to membership you are ineligible. If you can afford it go into a hospital to prepare for your L.O.S. certificate. The British Lying-in Hospital, Endell Street, W.C., charges £22 10s. for three months. At the East End Mothers' Home, 396, Commercial Road, E., the fee is £15 15s. See answer "L.O.S."

*Cancer.*

(133) What is the treatment of cancer? What are a nurse's duties with regard to disinfectants? How is it infectious? What precautions should a nurse take?—*Saucy.*

"Saucy" asks us to answer in a line a volley of questions upon which volumes have been and will be written. It is only recently that it has been thought to be infectious at all—and that not from person to person, but from dwelling-place to inmate. The precautions a nurse should take therefore are to keep herself in first-rate health, her patient and surroundings scrupulously clean, and to observe the usual rules for using disinfectants, the choice of which and of the treatment is usually decided by the medical man attending the case, and not by the nurse.

*Recognised Training School.*

(134) Will you say whether the Infirmary, Burton-on-Trent, is a recognised training school for nurses or not?—*Prebend.*

It is quite impossible to say which are recognised training schools and which are not until the Local Government Board schedules a list. The safest plan, when there is any doubt about the matter, is to write up to the Secretary (Whitehall) and ask. The General Infirmary at Burton-on-Trent possesses only 54 beds, and grants only two years' certificates. It is not probable, therefore, that the Local Government Board would recognise the training as qualifying the possessor for the post of superintendent in a Poor-law institution.

*Bandaging.*

(135) Can you recommend an inexpensive book on bandaging? and (2) one on the management of skin diseases from a nurse's point of view?—*C. P.*

You would probably find "Surgical Ward Work," by Miles (Scientific Press), suit you. (2) There does not seem to be any special book on nursing skin diseases.

*Pensions.*

(136) I understand that you can sometimes assist deserving people who have obtained their living as nurses to some little pension or help. I am interested in a respectable person who has been a nurse for 34 years, and who is now 67 years of age. She went to a lady who afterwards died, and she has remained at the same place as cook-housekeeper. She now feels the work is too much for her. Would it be possible to get her some weekly help?—*E. C.*

The position of aged nurses who have not had the modern training is one of peculiar hardship. There is no provision for them as nurses, for to benefit by the Royal National Pension Fund and the Junius Morgan Benevolent Fund it is necessary to be a member and to have paid yearly subscriptions. The Trained Nurses' Annuity Fund requires candidates to have worked for three years in a hospital. There only remains, therefore, charities common to all. You will find a list in Burdett's "Hospitals and Charities" (Scientific Press, 5s.) The secretary, the Aged Pilgrim's Society, 83, Finsbury Pavement, E.C.; or the secretary, the Universal Beneficent Society, 15, Soho Square, W., would inform you if your candidate were eligible for help from their funds, and, if so, how to apply.

*Certificate.*

(137) Please tell me how I can get into a provincial hospital for six or more months' training, and where to apply. I have not been in a hospital, but have had private experience for five years. I want a certificate without having to pay a premium.

It is quite impossible to get a certificate in general nursing in less than a year, and even that certificate will not open the door to the best workhouse appointments. Your best plan would be to advertise for what you want. Many hospitals pay their nurses from the beginning of their probation, if they enter for the three years' certificate.

## Figures and Tables

**Figure f1:**
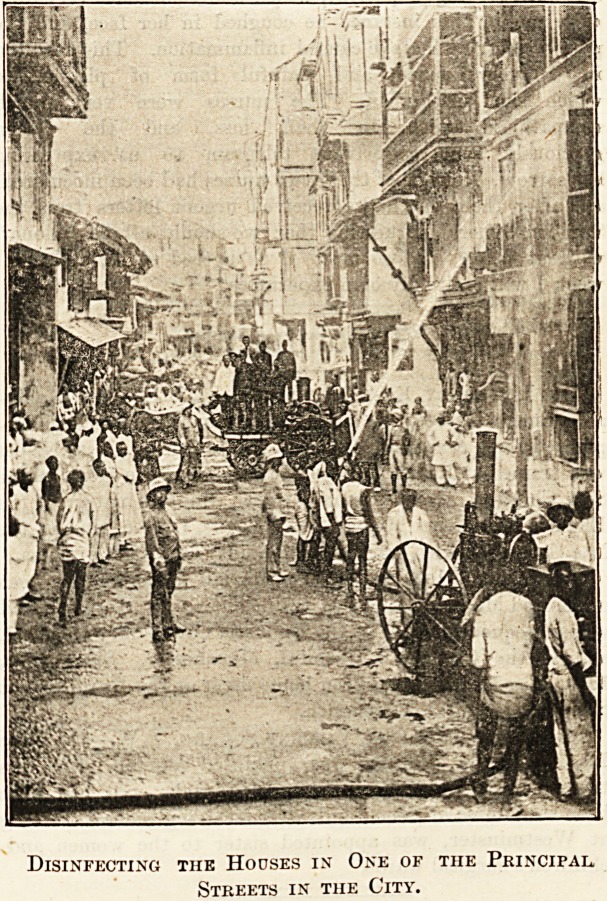


**Figure f2:**
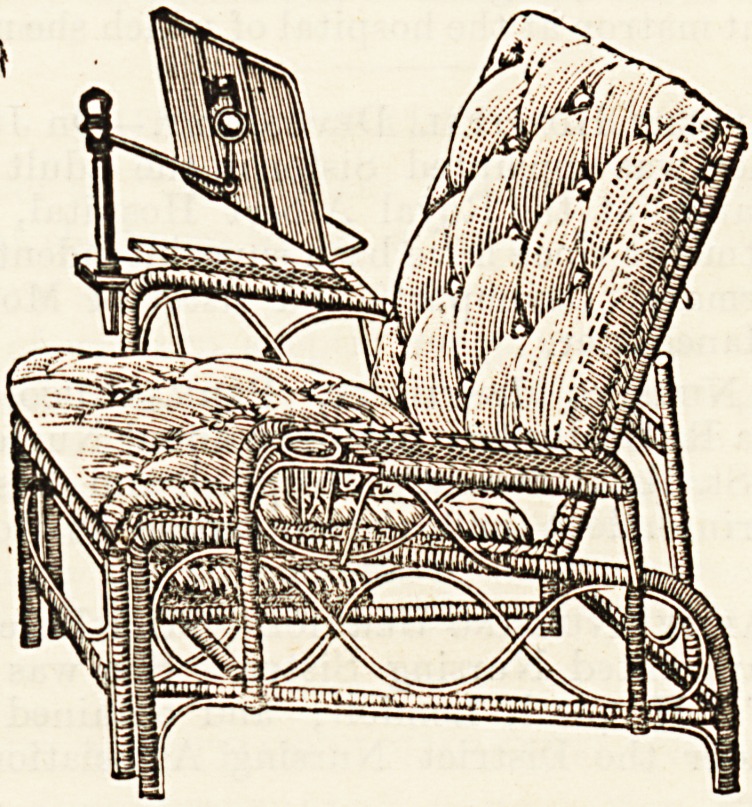


**Figure f3:**
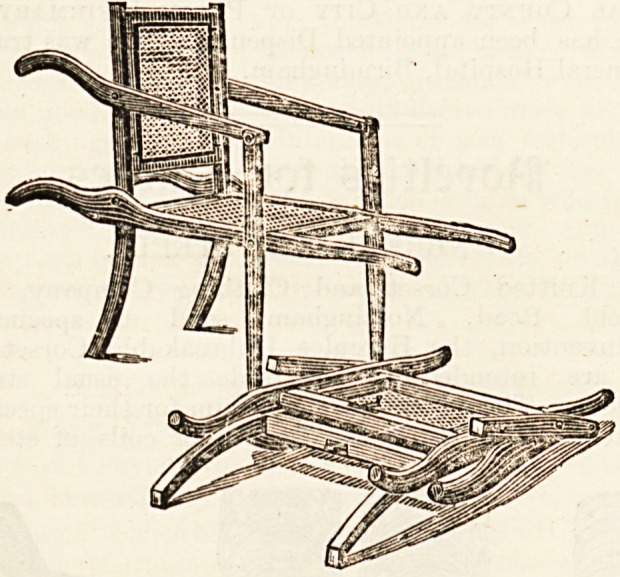


**Figure f4:**
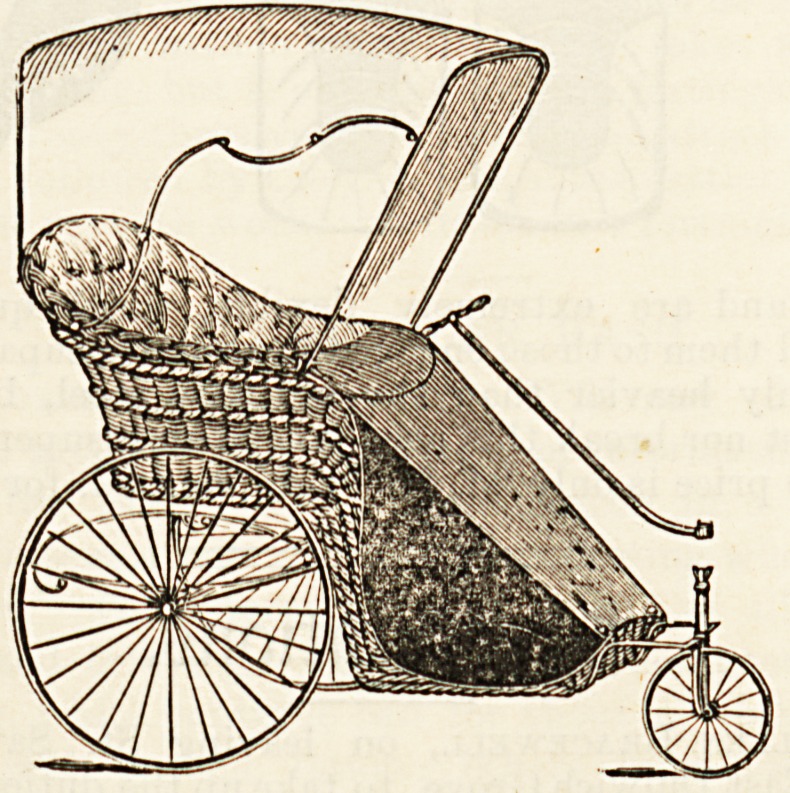


**Figure f5:**